# A CNN model for early detection of pepper Phytophthora blight using multispectral imaging, integrating spectral and textural information

**DOI:** 10.1186/s13007-024-01239-7

**Published:** 2024-07-29

**Authors:** Zhijuan Duan, Haoqian Li, Chenguang Li, Jun Zhang, Dongfang Zhang, Xiaofei Fan, Xueping Chen

**Affiliations:** 1 College of Mechanical and Electrical Engineering, Hebei Agricultural University, Baoding, 071000 China; 2 State Key Laboratory of North China Crop Improvement and Regulation, Hebei Agricultural University, Baoding, 071000 China; 3 College of Horticulture, Hebei Agricultural University, Baoding, 071000 China

**Keywords:** Multispectral imaging, Pepper Phytophthora blight, Early disease detection, Spectral textural fusion, Machine learning, Convolutional neural network

## Abstract

**Background:**

Pepper Phytophthora blight is a devastating disease during the growth process of peppers, significantly affecting their yield and quality. Accurate, rapid, and non-destructive early detection of pepper Phytophthora blight is of great importance for pepper production management. This study investigated the possibility of using multispectral imaging combined with machine learning to detect Phytophthora blight in peppers. Peppers were divided into two groups: one group was inoculated with Phytophthora blight, and the other was left untreated as a control. Multispectral images were collected at 0-h samples before inoculation and at 48, 60, 72, and 84 h after inoculation. The supporting software of the multispectral imaging system was used to extract spectral features from 19 wavelengths, and textural features were extracted using a gray-level co-occurrence matrix (GLCM) and a local binary pattern (LBP). The principal component analysis (PCA), successive projection algorithm (SPA), and genetic algorithm (GA) were used for feature selection from the extracted spectral and textural features. Two classification models were established based on effective single spectral features and significant spectral textural fusion features: a partial least squares discriminant analysis (PLS_DA) and one-dimensional convolutional neural network (1D-CNN). A two-dimensional convolutional neural network (2D-CNN) was constructed based on five principal component (PC) coefficients extracted from the spectral data using PCA, weighted, and summed with 19-channel multispectral images to create new PC images.

**Results:**

The results indicated that the models using PCA for feature selection exhibit relatively stable classification performance. The accuracy of PLS-DA and 1D-CNN based on single spectral features is 82.6% and 83.3%, respectively, at the 48h mark. In contrast, the accuracy of PLS-DA and 1D-CNN based on spectral texture fusion reached 85.9% and 91.3%, respectively, at the same 48h mark. The accuracy of the 2D-CNN based on 5 PC images is 82%.

**Conclusions:**

The research indicates that Phytophthora blight infection can be detected 48 h after inoculation (36 h before visible symptoms). This study provides an effective method for the early detection of Phytophthora blight in peppers.

**Supplementary Information:**

The online version contains supplementary material available at 10.1186/s13007-024-01239-7.

## Background

Peppers are an important horticultural crop cultivated worldwide, with fruits rich in carotenoids and vitamins A, B, and C [1]. Peppers are often threatened by Phytophthora blight during production. Phytophthora blight is a fungal disease caused by Phytophthora oomycetes [2] that can rapidly invade plants, leading to yellowing and rotting of leaves and even death of the entire plant, severely affecting the yield and quality of peppers [3]. In the treatment of Phytophthora blight in peppers, the earlier the treatment, the higher is the cure rate. When the disease first begins to infect pepper plants, the number of pathogens is relatively low and plant resistance is not yet completely suppressed [4]. Therefore, early detection of pepper Phytophthora blight and taking corresponding measures are of great importance for agricultural production.

Traditionally, the most common method for identifying Phytophthora blight in peppers is visual inspection. However, Phytophthora initially infects the root base, which is difficult to observe as it is in the soil, and the leaves show no symptoms at the early stages. Therefore, it is difficult to determine whether the pepper is infected with Phytophthora blight in the early stages [5, 6]. Although fluorescence in situ hybridization (FISH), enzyme-linked immunosorbent assay (ELISA), and polymerase chain reaction (PCR) are relatively accurate detection methods, these techniques are destructive, expensive, and unsuitable for large-scale rapid detection. Thus, accurate, rapid, and non-destructive techniques are essential for the early identification of Phytophthora blight in peppers [7, 8]. To meet this need, researchers have been continually exploring and developing new detection technologies.

In recent years, spectral imaging technology has demonstrated remarkable capabilities in providing information on plant health and predicting disease onset [9]. Multispectral imaging technology can detect subtle changes in crop leaves by capturing spectral reflectance at different wavelengths, enabling the determination of biochemical and physiological responses such as leaf pigment composition and moisture content. Taking advantage of this, spectral images combined with chemometrics have been successfully applied for disease identification in various crops. Sinha et al. [10] evaluated the applicability of visible and near-infrared (NIR) technology for detecting Grapevine leafroll-associated virus 3 and found that spectral features in specific wavelength regions could accurately distinguish between healthy and infected leaves, achieving overall classification accuracies between 75 and 99% using a quadratic discriminant analysis classifier. Gu et al. [11] established an early non-destructive detection method for Tobacco tomato spotted wilt using three wavelength selection methods and four machine learning techniques. The accuracy rate of the boosted regression trees model based on spectral features extracted using the sequential projection algorithm (SPA) was 85.2%. Karadağ et al. [12] detected Fusarium wilt in peppers using spectral reflectance data from 350 to 2500 nm combined with three machine learning algorithms: artificial neural network, naive Bayes, and k nearest neighbors (KNN). The KNN method yielded the best classification performance with an accuracy of 99%. The above studies show that the information of spectral dimension of hyperspectral images performs well in the early detection of diseases; however, most studies have not considered the correlation between spatial information and diseases.

Two-dimensional spatial information (texture) in multispectral images also includes disease-related information [13]. Textural features describe the spatial distribution of brightness levels in adjacent pixels and reflect object appearance characteristics, such as color, texture, and shape [14]. Zhu et al. [15] used the gray-level co-occurrence matrix (GLCM) to extract textural features and established different machine learning models to identify healthy and diseased tobacco leaves at different stages, with a classification accuracy of 93.33% for the back propagation neural network. Xuan used SPA to extract effective wavelengths and GLCM to extract textural features; the accuracy of the combination of these two features after partial least squares discriminant analysis (PLS-DA) was 91.4% [16]. In recent years, deep learning has been widely applied in image classification owing to its ability to automatically learn abstract features, strong representation capabilities, and modeling abilities. Convolutional neural networks (CNN) have significant advantages for large-scale image processing. Cui et al. [17] established a dual-channel convolutional neural network model (combining three-dimensional-CNN and one-dimensional (1D)-CNN), for early detection of apple decay diseases.

While combining spectral features and textural features with machine learning and deep learning algorithms for the early detection of Phytophthora blight in peppers is feasible, this field still faces some challenges and potential issues. First, Phytophthora blight primarily affects the root base, where the pathogen forms mycelia and sporangia in the xylem, leading to xylem vessel blockage. This reduces the water and nutrients transported to the leaves. The root base is the initial infection site, and changes in the leaves are mainly indirect physiological responses due to root damage [18]. Compared to other plant diseases that primarily infect leaves, the physiological and biochemical changes in leaves caused by Phytophthora blight are relatively minor, making early detection challenging. This places higher demands on the precision of image acquisition equipment and the suitability of feature selection methods and models.

Therefore, this study explores advanced feature selection algorithms and develops models suitable for disease detection using high-resolution multispectral imaging technology to improve classification accuracy and achieve early detection of Phytophthora blight in peppers. The specific objectives are: (1) Extract spectral and textural information from multispectral images of healthy and diseased pepper leaves; (2) Use principal component analysis (PCA), SPA, and genetic algorithm (GA) for spectral texture feature selection; perform weighted summation on 19-channel multispectral images based on PCA to obtain 5 principal component images.; (3) Based on spectral data, combined with different features (single spectral and spectral textural fusion) and different models (PLS-DA and 1D-CNN), evaluate their performance in the early detection of Phytophthora blight in peppers. Based on PCA fusion images, establish a two-dimensional (2D)-CNN model to further evaluate the detection characteristics of different models.

## Materials and methods

### Sample preparation

This experiment selected the pepper material "16–217" and the Phytophthora strain ph3, provided by the Key Laboratory of Vegetable Germplasm Innovation and Utilization in Hebei Province. The pepper plants of this variety are 60–80 cm tall, with cylindrical fruits that taper at the tip, measuring 15–20 cm in length. The fruits are smooth and colorful, green when unripe, and red when mature. In a dedicated nursery environment, 149 pepper seedlings were cultivated, 76 in the healthy group and 73 in the diseased group. Twenty-one days after pepper planting, the infected pepper plants were artificially inoculated under simulated natural environmental conditions, and the healthy pepper plants in the same growth cycle were used as the control. Using the root drenching inoculation method, a glass rod was used to make a small hole 3 cm deep and 1 cm from the base of the plant stem. A syringe was then used to inject 5 ml of a zoospore suspension with a concentration of 1 × 10^7^ spores/ml into the hole. Afterwards, the healthy and infected pepper plants were stored in different greenhouses at the same temperature (25 °C) and relative humidity (90%).

### Multispectral imaging systems and acquisition

In this study, VideometerLab 4 (Videometer, Herlev, Denmark) spectral imaging equipment was used for image acquisition, which integrates lighting, cameras, and computer technology with advanced digital image analysis and data statistics capabilities. It can be used for the rapid and effective determination of the surface color, texture, and chemical composition with an image resolution of 2192 × 2192. Spectral images with 19 wavelengths can be obtained (365 nm,405 nm,430 nm,450 nm,470 nm,490 nm,515 nm,540 nm,570 nm,590 nm,630 nm,645 nm,660 nm,690 nm,780 nm,850 nm,880 nm,940 nm,970 nm), including visible light, ultraviolet, and NIR imaging.

**Fig. 1:**
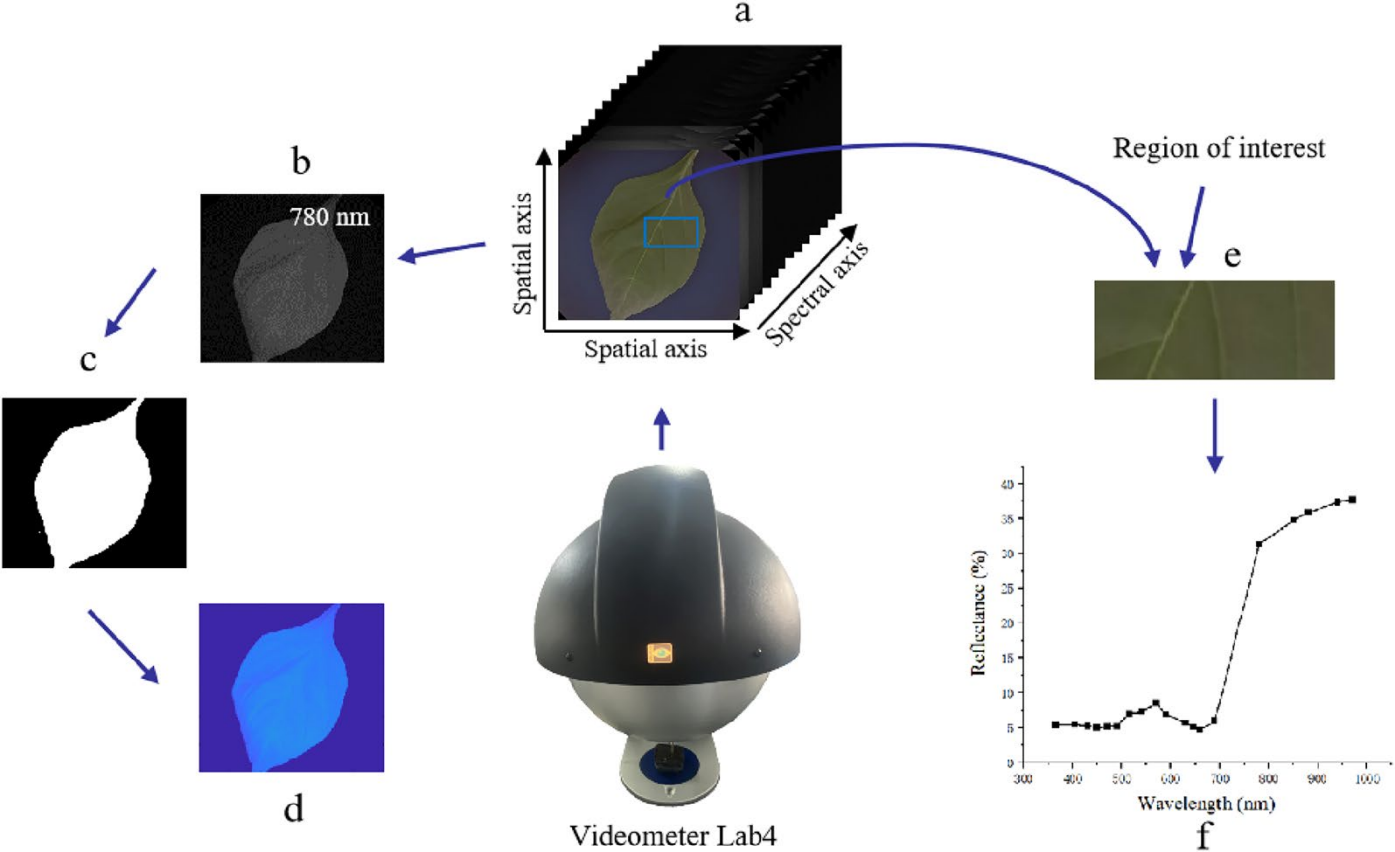
Multispectral image acquisition and processing. **a** 19-channel multispectral image; **b** image at 780 nm; **c** binarized image; **d** result of pixel-wise multiplication of b and c; **e** 19-channel spectral reflectance; **f** selected ROI

The spectral imager was focused to ensure that the acquired spectral image was clear and not deformed. The descending height of the multispectral imager was adjusted to 20 cm and the height was increased to 90 cm. Pepper leaves were collected in vivo before and at 48, 60, 72, and 84 h after Phytophthora fungus. Pepper plants were placed upside down in a closed diffusion sphere, and one complete and clear leaf was selected and placed on a sample table. Click F12 was used to collect multi-spectral images, and 19 reflectance images were obtained at different wavelengths. Figure 1 shows the acquisition and processing of the multispectral images. The right-hand section shows the extraction of spectral reflectance by selecting the region of interest (ROI). Since the image at the 780 nm is clearer, it is beneficial for extracting textural features. The left-hand section shows the extraction process of textual features by selecting images with a 780 nm.

### Data extraction

Using the VideometerLab software, the ROI was selected from the acquired multispectral images. The spectral data were analyzed based on the ROI. Three ROI of similar size were selected from the infected and healthy leaves, and the average wavelength reflectance was obtained by averaging the reflectance of each wavelength in the target region.

During infection, the surface texture of pepper leaves undergoes significant changes. Texture reflects the biophysical and biochemical characteristics of plants and is directly or indirectly related to plant growth. The GLCM measures the co-occurrence relationships between the gray levels of each pixel in an image, yielding four features: contrast, energy, homogeneity, and correlation [19]. Textural information was extracted from the entire image area, and each textural feature was interpreted as follows:

1 $$Contrast\, = \,\sum\limits_{i = 1}^{N} {\sum\limits_{j = 1}^{N} {\left( {i - j} \right)} }^{2} P\left( {i,j} \right)$$

2 $$Energy\, = \,\sum\limits_{i = 1}^{N} {\sum\limits_{j = 1}^{N} {P\left( {i,j} \right)} }^{2}$$

3 $$Homogeneity\, = \,\sum\limits_{i = 1}^{N} {\sum\limits_{j = 1}^{N} {\frac{{P\left( {i,j} \right)}}{{1 + \left( {i-j} \right)^{2} }}} }$$

4 $$Correlation\, = \,\frac{{\sum\limits_{i = 1}^{N} {\sum\limits_{j = 1}^{N} {\left( {i,j} \right)\,P\left( {i,j} \right) - \mu_{i} \mu_{j} } } }}{{\sigma_{i} \sigma_{j} }}$$

where P(*i*,*j*) is the element in the *i*-th row and *j*-th column of the GLCM, representing the probability of the gray levels *i* and *j* occurring together. *μ*_*i*_ and *μ*_j_ are the mean values, and *σ*_*i*_ and *σ*_*j*_ are the standard deviations corresponding to the rows and columns of P(*i*,*j*), respectively.

Local binary patterns (LBPs) generate binary codes to reveal textural information by comparing the gray values between each pixel in the image and its neighboring pixels. Parameters LBP1 to LBP59 were extracted from the LBP, and parameters with a value of zero were eliminated. Sixteen features remained.

### Data preprocessing

The Savitzky–Golay (SG) filter and standard normal variate (SNV) were used to pretreat the original spectrum to reduce the influence of environmental factors and internal sample variation on the spectral data. The SG filter is based on the principle of local least squares, smoothing data by fitting polynomials, effectively reducing noise and preserving useful features in the signal as much as possible [20]. The SNV was used to calibrate the systematic biases caused by factors such as particle size and surface scattering among the samples. This improves the comparability between different samples by centering each sample's spectrum on the mean and dividing it by the standard deviation. This was calculated using the following formula:

5 $$X_{SNV} = \,\frac{{x - \overline{x} }}{\sigma }$$

6 $$\sigma = \,\frac{{x - \overline{x} }}{{\sqrt {\frac{{\sum\limits_{k = 1}^{m} {\left( {x_{k} - \overline{x} } \right)} }}{{\left( {m - 1} \right)}}} }}$$

where *m* represents the number of spectral textural features, k = 1, 2, ..., m, and *x* is the spectral textural feature value.

### Feature selection

Multispectral images comprise high-dimensional data with redundant information that can cause instability when directly applied to classification. Selecting specific feature wavelengths containing critical information for distinguishing between diseased and healthy leaves can improve the stability and computational efficiency of a model. Feature selection was conducted using three methods: PCA, GA, and SPA. PCA is an unsupervised linear dimensionality-reduction technique that transforms potentially correlated high-dimensional variables into linearly uncorrelated variables, known as principal components (PCs), through orthogonal transformation. PCA reduces the data dimensions while retaining the most information possible. The new orthogonal variables produced in the PCA transformation are linear combinations of all original variables [21]. GAs are optimization algorithms based on the theories of natural selection and genetics [22] that find the optimal solution to the problem by simulating the evolutionary process of the natural world. In feature selection, they iterate to search for the optimal feature subset to improve classification performance. Konak et al. [23] introduced the basic principles of GA. The SPA is a forward variable selection method that begins by selecting one variable and then introduces another variable in each iteration through a projection procedure. The newly selected variable has a maximum projected value on the orthogonal subspace of the previously selected variable [24]. Araújo et al. [25] described the SPA process in detail.

### Establishment of classification model

Different machine and deep learning models (PLS_DA, 1D-CNN, and2D-CNN) were used for the early detection of Phytophthora pepper and compared. PLS_DA was conducted using PyCharm Community Edition (JetBrains, Prague, Czech Republic), 1D-CNN, and 2D-CNN in MATLAB R2021a (MathWorks, Natick, MA, USA).

#### Classification models based on spectral data

**Fig. 2:**
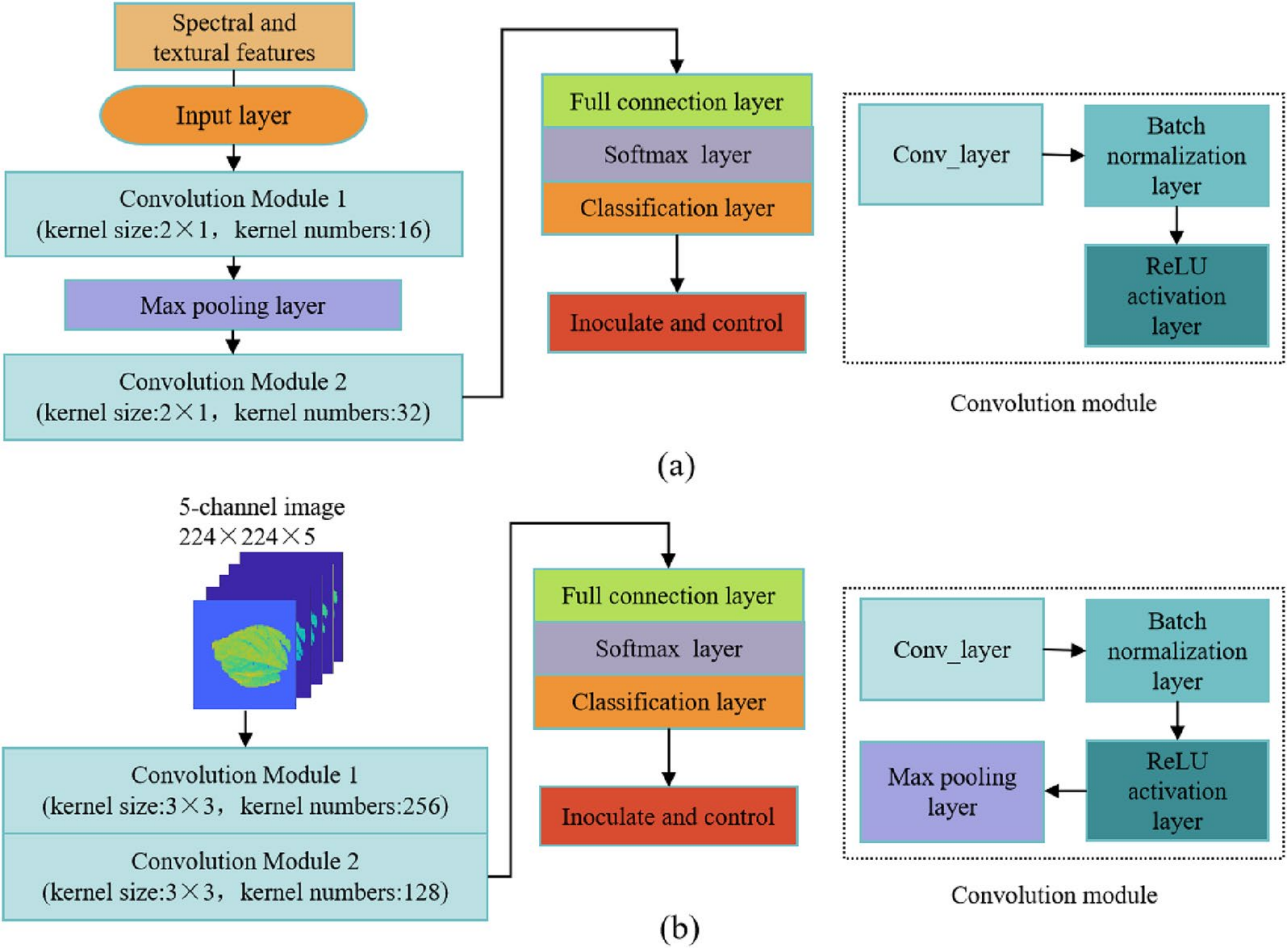
Model structure diagram; **a** structure of the 1D-CNN model; **b** structure of the 2D-CNN model

PLS_DA is a supervised learning method that integrates the concepts of partial least squares regression and discriminant analysis. PLS_DA classifies samples by identifying the relationships between explanatory variables, such as spectral data, and response variables, such as the health of the sample. This method is particularly suitable for processing multicollinear high-dimensional data [26]. A CNN is a deep learning model that performs well in image recognition and classification [27]. The CNN model automatically learns features through a series of convolution, activation, pooling, and fully connected layers [28–30]. To solve the problems of disappearing gradients, fast convergence, and anti-overfitting, batch normalization (BN) layers and Rectified Linear Unit activation functions were introduced during training. The BN layer accelerates the training by regularizing the intermediate layer output [31]. Figure 2(a) shows the structure of the 1D-CNN, which consists of an input layer, two convolution layers, two activation layers, two batch normalization layers, one maximum pooling layer, one fully connected layer, and one classification layer. Finally, softmax was used to complete the disease diagnosis.

#### Classification model based on multispectral images

Using the multispectral image as input, a 2D-CNN model was established, and the input layer of the model was a five-channel image of 224 × 224 pixels. Considering the limitation of the amount of data, our CNN model was developed manually to avoid overfitting. The final model comprised 12 layers, including one input layer, two convolution layers, two activation layers, two normalization layers, two pooling layers, one fully connected layer, one softmax layer, and a classification layer. Figure 2(b) shows the 2D-CNN model for the early detection of Phytophthora pepper. The convolution module of the model comprises convolution, normalization, and activation layers. In this experiment, the size of the convolution kernel was set to 3 × 3. Normalization was used for batch normalization, the activation layer was used as the Leaky-related activation function, and the pooling layer was used for maximum pooling.

### Model evaluation and validation

**Fig. 3:**
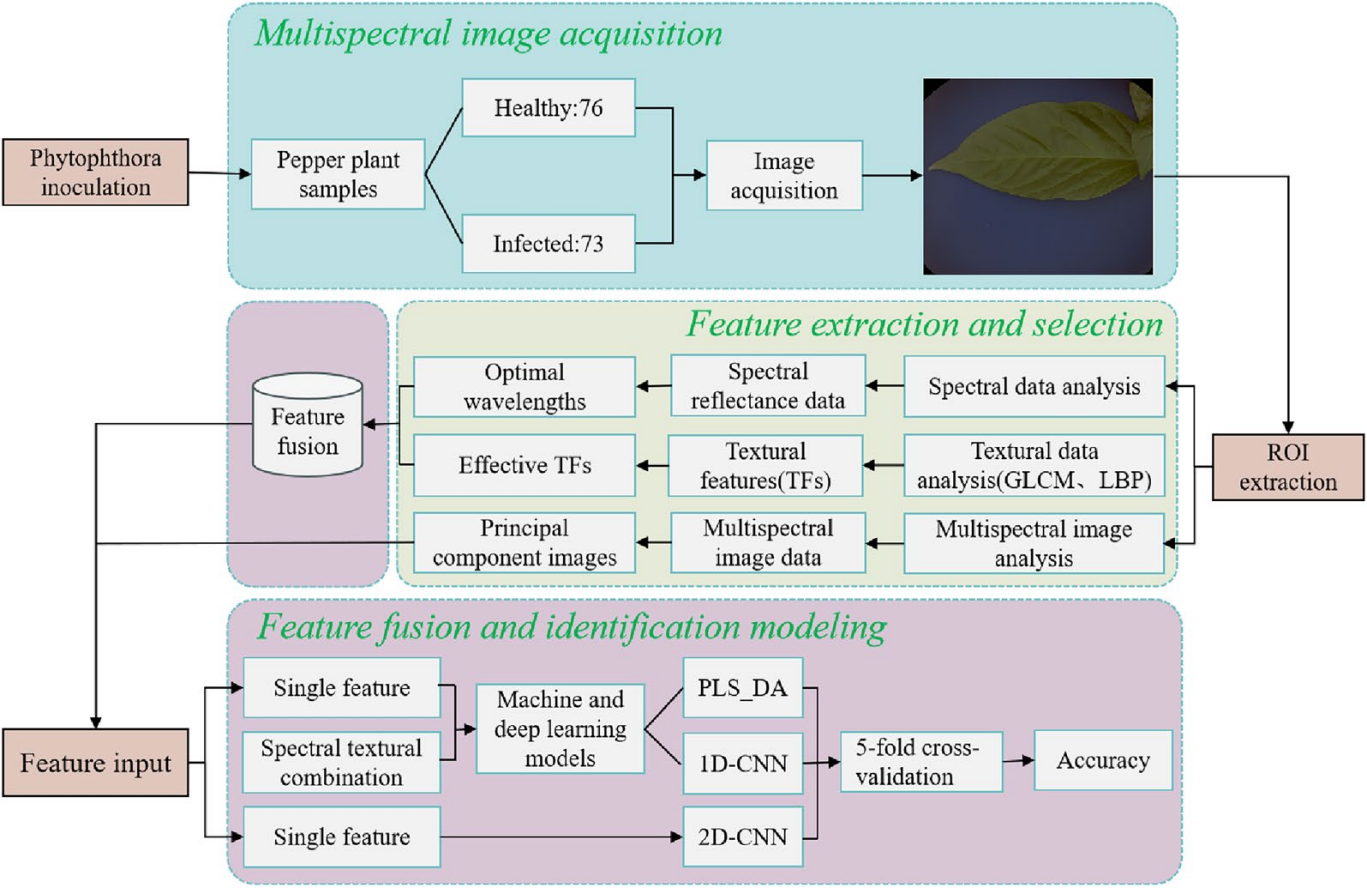
Pepper Phytophthora blight early detection flowchart

Faced with a limited number of samples, this study adopted a five-fold cross-validation method to evaluate model performance [32]. Unlike the traditional method of splitting a dataset into two sets for model training and performance verification, it divides all samples into a preset number (N) of groups, where the N-1 group is used for model training, and the remaining samples are used for validation. The training and verification processes were performed N times, and each group was used as the verification group. Model performance was evaluated by recording the average value of the evaluation index. In general, N is defined as five, that is, five times cross-validation. The primary steps of this study are shown in the flowchart in Fig. 3.

## Results

### Spectral characteristics of healthy and diseased leaves

**Fig. 4:**
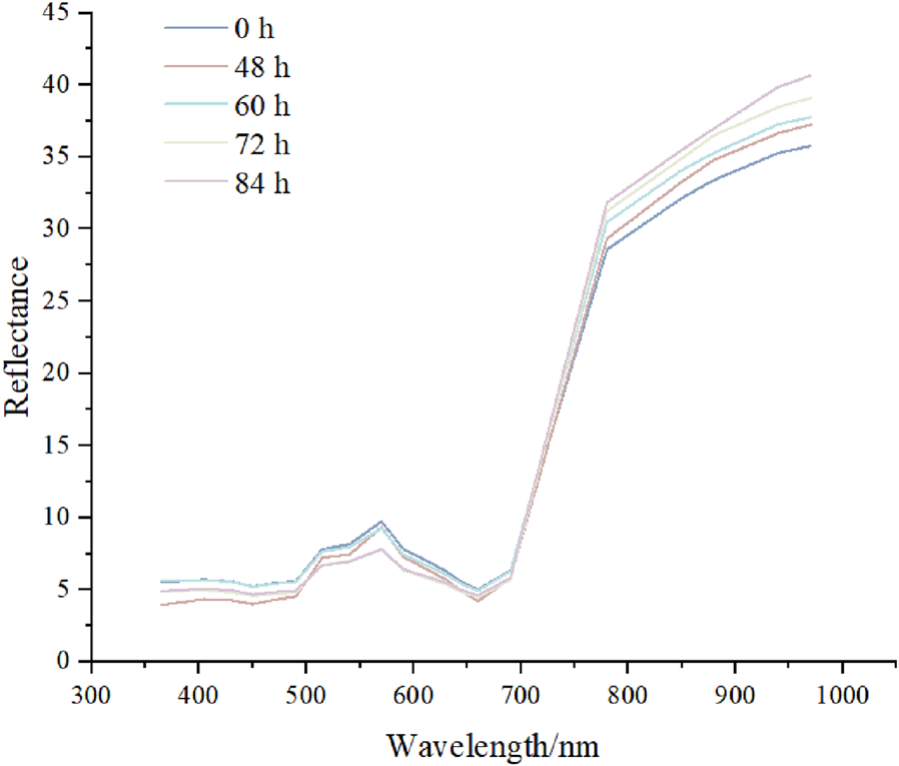
Spectral changes of pepper leaves at different times

Figure 4 shows the average spectral reflectance before inoculation and 48, 60, 72, and 84 h after inoculation, where the absorption peaks or troughs of the spectral curve reflect the variation in the spectrum, which is related to the physical and chemical properties of the leaves [33, 34]. 400–690 and 780–1000 nm were in the visible and NIR regions, respectively. The curves show that the visible reflectance of both samples was lower than that in the NIR region. In the 470–645 nm wavelength range, the reflectance of healthy samples is the highest, significantly exceeding that of diseased samples. The reflectance is the lowest 84 h after inoculation. In the NIR region, however, the reflectance of healthy samples is actually the lowest. As the duration of the disease increases, the reflectance shows an upward trend.

### Effective feature selection

**Fig. 5:**
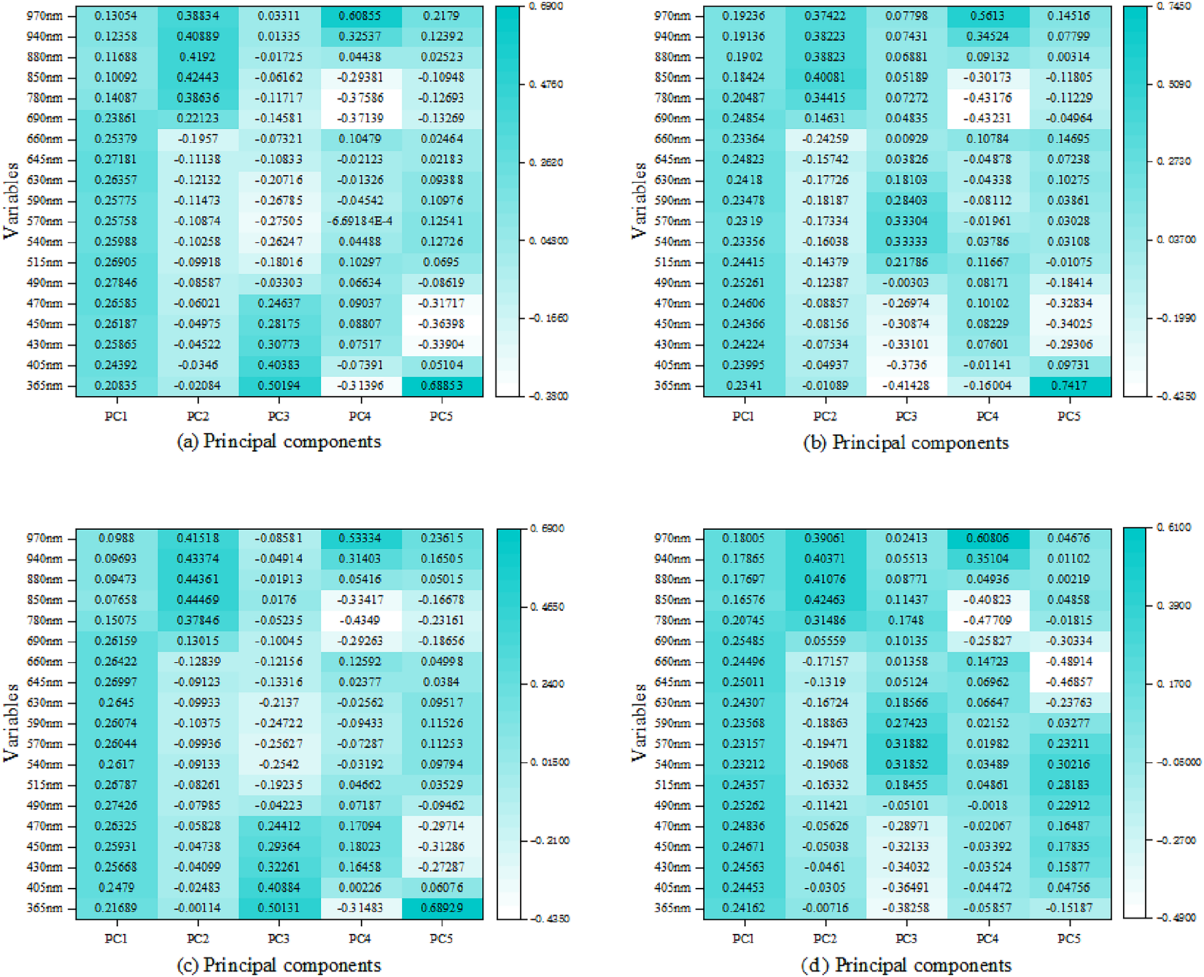
**a** PCA coefficient matrix plot at 48 h; **b** PCA coefficient matrix plot at 60 h; **c** PCA coefficient matrix plot at 72 h; **d** PCA coefficient matrix plot at 84 h

PCA was performed on the data at 48, 60, 72, and 84 h after SG preprocessing. Nineteen variables were replaced with composite variables, accounting for approximately 25% of the original variables, and five PCs were extracted. Figure 5 shows the load matrix. At 48 h, the variance percentages of PC1 and PC2 were 64.15 and 24.83%, respectively, and the variance sum of these PCs accounted for 89.98% of the total variance, retaining the information of the original wavelength. At 60, 72, and 84 h, the variance proportions of PC1 and PC2 were the highest. The highest absolute value of the coefficients of each variable indicated that the PC integrated variables with high absolute values. Therefore, it can be concluded that the 48h PC1 primarily integrated the wavelengths at 470, 490, 515 and 645 nm, and PC2 primarily integrated the wavelengths at 850, 880, 940 and 970 nm. The integrated wavelength information of the first and second PCs at 60, 72, and 84 h is shown in Table 1. The results of the feature wavelength selection by SPA and GA at 48, 60, 72, and 84 h are shown in Supplementary Figure A1 and A2.

**Table 1:** The characteristic wavelengths of the primary synthesis of PCs obtained using PCA

Time/h	Effective features
48	470 nm, 490 nm, 515 nm, 645 nm, 850 nm, 880 nm, 940 nm, and 970 nm
60	470 nm, 490 nm, 645 nm, 690 nm, 850 nm, 880 nm, 940 nm, and 970 nm
72	490 nm, 515 nm, 630 nm, 645 nm, 850 nm, 880 nm, 940 nm, and 970 nm
84	470 nm, 490 nm, 645 nm, 690 nm, 850 nm, 880 nm, 940 nm, and 970 nm

Owing to the large differences between spectral and textural feature data, SNV was used for data preprocessing to eliminate dimensional differences between different features or samples so that the data are in a unified scale range. After SNV pretreatment, spectral textural data were selected using SPA and GA at 48, 60, 72, and 84 h. The results are summarized in Table A1. Using PCA, 39 variables were replaced with synthetic variables, accounting for approximately 30% of the original variables, and 12 PCs were extracted.

### Early diagnosis model based on spectral features

Using PCA, SPA, and GA to select effective feature wavelengths, PLS-DA and 1D-CNN were employed to classify diseased and healthy leaves at 48, 60, 72, and 84 h post-inoculation. In the first six rows of Table 2, the SPA, GA, and PCA spectra represent the single spectral feature inputs. The accuracy of PCA combined with PLS-DA and 1D-CNN showed an upward trend across the four time points, reaching > 90% after 60 h. This indicates the feasibility of using spectral data for the early detection of pepper Phytophthora blight. The GA combined with PLS-DA achieved the highest accuracy at 48 h; however, the accuracy decreased at 72 h. This is because the extracted feature wavelengths did not include all the wavelengths influenced by disease characteristics, resulting in random spectral fluctuations and affecting model recognition accuracy. In addition, these models can be considered fast because their training processes can be completed within a few seconds.

**Table 2:** Classification accuracy of five-fold cross-validation of single spectral features and spectral textural fusion features

Input features	Model	Overall accuracy (%)			
		48 h	60 h	72 h	84 h
SPA spectrum	PLS_DA	79.9%	90.6%	89.2%	88.6%
	1D-CNN	77.2%	91.3%	91.2%	90.4%
GA spectrum	PLS_DA	86%	90%	88.6%	89%
	1D-CNN	82%	86.7%	83.2%	93.9%
PCA spectrum	PLS_DA	82.6%	92.6%	92.6%	93.3%
	1D-CNN	83.3%	90.6%	93.3%	95.1%
SPA spectrum–texture	PLS_DA	82.6%	91.9%	90.6%	90.4%
	1D-CNN	80.6%	87.3%	92%	96.7%
GA spectrum–texture	PLS_DA	86.7%	91.3%	91.9%	92%
	1D-CNN	82.7%	86.5%	92%	92.4%
PCA spectrum–texture	PLS_DA	85.9%	92%	93.9%	94.5%
	1D-CNN	91.3%	92.6%	92.6%	94.7%

### Early diagnostic model based on spectral textural fusion features

To further explore the role of changes in internal components and external properties in the early detection of pepper Phytophthora blight, spectral and textural features of multispectral images were combined to establish PLS-DA and 1D-CNN models. In the last six rows of Table 2, the SPA, GA, and PCA spectra–textures represent the fused spectral and textural feature inputs. The data processed through PCA feature selection and then applied to the 1D-CNN achieved a classification accuracy of 91.3% at 48 h, with an increasing trend at 60, 72, and 84 h, achieving accuracies of 92.6, 92.6, and 94.7%, respectively.

### Early classification models based on multispectral images

**Fig. 6:**
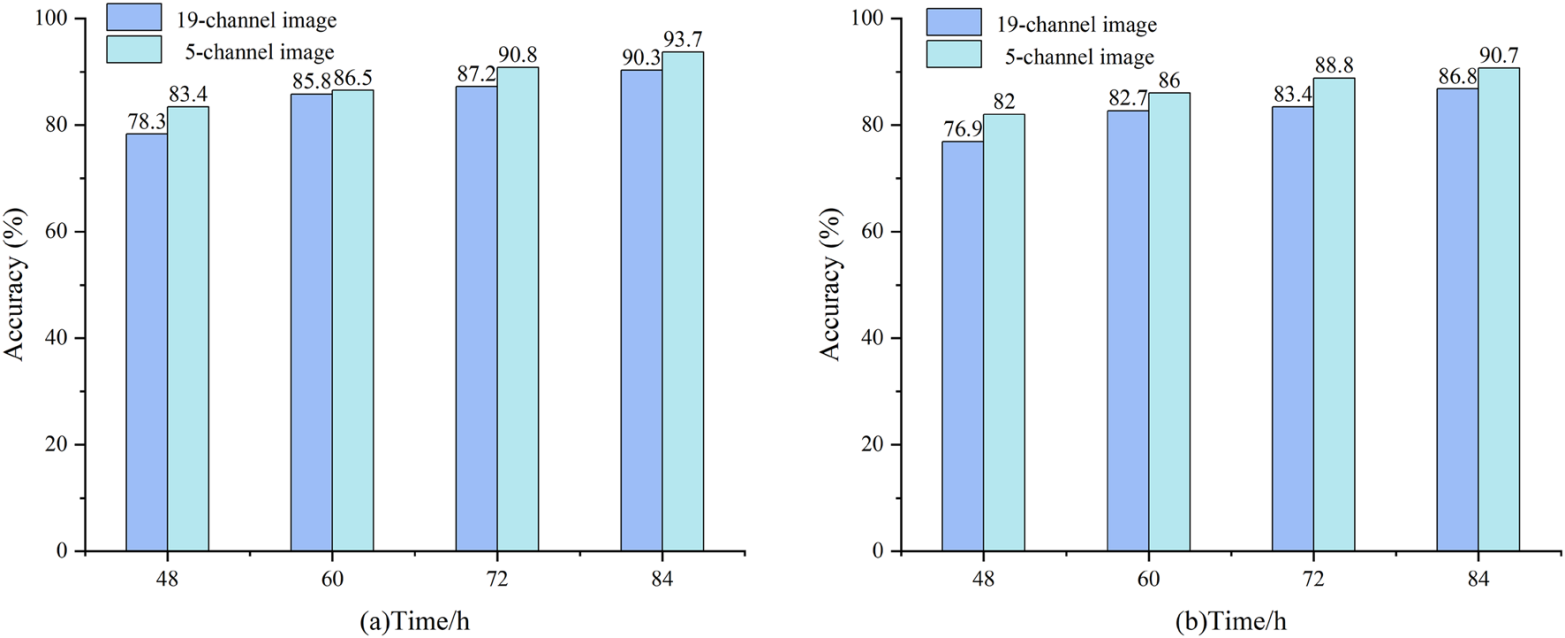
Comparison of classification accuracy for 2D-CNN models based on different features; **a** five-fold cross-validation results on the training set; **b** five-fold cross-validation results on the test set

Among the models established based on spectral data, the classification accuracy after PCA dimensionality reduction was the highest. Therefore, the five PC coefficients extracted by the PCA were weighted and summed to obtain five new PC images. Each PC image is composed of the product of the corresponding PC coefficient and the pixel value of the original multispectral image, reflecting the feature information of the original data in the direction of the PC. This PCA-based method can effectively extract and compress key information from the original multispectral data, which is convenient for subsequent analysis and processing. The classification results of the 19- and 5-channel images after PCA dimensionality reduction using the 2D-CNN model are shown in Fig. 6. The accuracies of the five-channel image on the 48, 60, 72 and 84h training sets were 82, 86, 88.8, and 90.7%, respectively.

## Discussion

### Spectral analysis

The overall trends of the spectral reflectance curves for healthy and infected leaves are very similar, but differences can be observed in the 470–645 nm and 780–970 nm bands. The key wavelengths extracted by PCA include 470 nm, 490 nm, 515 nm, 630 nm, 645 nm, 850 nm, 880 nm, 940 nm, and 970 nm, covering the blue, green, and NIR bands. In the 470–645 nm range, which typically refers to the blue and green bands, healthy leaves contain higher chlorophyll levels. Chlorophyll weakly absorbs green light (approximately 500–570 nm), so healthy leaves have higher reflectance in the green light band. However, when leaves are infected by Phytophthora, chlorophyll content decreases, primarily because the pathogen disrupts the structure and function of chloroplasts, inhibiting photosynthesis. As chlorophyll content decreases, the reflectance of infected leaves in the green light band also changes [35]. The 780–970 nm band typically refers to the NIR band, which is mainly related to the internal structure and water content of the leaves. Healthy leaves have high reflectance in the NIR band because their internal cell walls and higher water content scatter and reflect a large amount of NIR light [36]. However, when leaves are infected by Phytophthora, the pathogen damages the cell structure of the leaves, leading to changes in the intercellular spaces and a decrease in water content. As the infection progresses, the water content of the leaves further decreases, and cell structure damage worsens, resulting in lower absorption and increased reflectance in the NIR band [37]. Additionally, when Phytophthora infects the plant from the root base and begins to spread upwards, it secretes toxins and enzymes that disrupt cell structure. This induces the leaves to produce defense substances, such as secondary metabolites and antimicrobial peptides, which also affect spectral reflectance [38].

### Classification performance comparison

This study combines single spectral features and spectral textural fusion features, using different algorithms and models to determine the most suitable method for early detection of pepper blight. The results show that the accuracy of the 1D-CNN based on single spectral features ranges from 83.3% to 95.1%, while the accuracy of the 1D-CNN based on spectral textural fusion features ranges from 91.3% to 94.7%. At 48 h, the accuracy increased from 83.3% with single-spectrum features to 91.3% with spectral textural fusion features. This indicates that combining spectral and textural features provides better classification results, as textural features also contain some disease-related information [39]. Regarding spectral textural fusion features, SNV is an effective spectral preprocessing method that not only improves the quality of spectral features but also enhances the recognizability of textural features. To extract spectral wavelengths that differentiate between healthy and infected leaves, SPA and GA are widely used feature selection algorithms [11, 15]. This study differs from previous research by introducing PCA for feature selection. The comparative results show that features extracted using PCA slightly improve model performance. The difference between PCA and SPA and GA lies in that PCA is a global dimensionality reduction method that systematically analyzes the overall structure of the data, selecting principal components based on the direction of the greatest variance in the data, thus retaining features with the most significant changes. In contrast, SPA incrementally selects features, which can be influenced by the selection order and local optima, and GA, while having high computational complexity, is less efficient than PCA.

The 1D-CNN model based on spectral textural fusion features achieved an accuracy of 91.3% at 48 h. Compared to previous studies on the early detection of other crop diseases using PLS_DA, for example, Xuan et al. [15] diagnosed wheat powdery mildew by combining spectral and textural features to establish a partial least squares regression model, achieving an accuracy of 85.7% at 48 h. In this study, the best result using the PLS_DA model was based on GA-selected features, with an accuracy of 86.7%, which is comparable to the model used by Xuan et al. However, the 1D-CNN model developed in this study demonstrated significantly better classification performance. This is mainly because 1D-CNN, compared to PLS_DA, has stronger nonlinear processing capabilities, automatic feature learning, and adaptability when handling multispectral data. These advantages make 1D-CNN exhibit higher accuracy and robustness in classification tasks, indicating that 1D-CNN may be more suitable for the early detection of pepper blight.

The 2D-CNN accuracy based on five PC images was only 82% at 48 h, which was not as high as that of 1D-CNN based on spectral and textural fusion features. However, this method can directly input the acquired image into the model for processing and does not require manual selection of the ROI to extract spectral data, which is simple and efficient and has great development prospects.

## Conclusions

Phytophthora blight is a serious hazard to pepper plants; therefore, early detection of the disease is necessary to take targeted measures to prevent its spread. The early stage is the best time to control Phytophthora disease, which is of great significance for improving the quality and yield of peppers and reducing agricultural production losses. This study explored the early detection of asymptomatic peppers inoculated with Phytophthora pepper , based on multispectral image technology, as a non-destructive testing method combined with machine learning. The primary conclusions are as follows:

Based on the spectral data, 19-wavelength spectral reflectance was extracted from multispectral images. Textural features were extracted using the GLCM and LBP algorithms. PCA, SPA, and GA were used to reduce the dimensionality of spectral and textural features, and two models, PLS-DA and 1D-CNN, were established. Among them, the 1D-CNN classification model based on the combination of spectral and textural features exhibited the best performance, and the accuracies at 48, 60, 72, and 84 h were > 90%. Based on the multispectral images, the five PC coefficients extracted from the PCA were weighted and summed to obtain five new 19-channel multispectral images, and a 2D-CNN model was established for classification, with results ranging from 82 to 90.7%.

Overall, the classification accuracy of the 1D-CNN model based on the combination of spectral and textural features was as high as 91.3% at 48 h of inoculation, indicating that Phytophthora infection could be detected 48 h after inoculation (36 h before visible symptoms). In future research, we will attempt to use multispectral technology combined with various feature-fusion methods to detect Phytophthora infections early under field conditions.

### Supplementary Information

Additional file 1: Figure results after SPA feature extraction;wavelength extracted at 48 h;wavelength extracted at 60 h;wavelength extracted at 72 h;wavelength extracted at 84 h. Figure results after GA feature extraction;wavelength extracted at 48 h;wavelength extracted at 60 h;wavelength extracted at 72 h;wavelength extracted at 84 h. Table Results of feature extraction using different methods.

## Data Availability

Please contact the corresponding author for data requests.

## Abbreviations

1D-CNN: One-dimensional convolutional neural network
2D-CNN: Two-dimensional convolutional neural network
BN: Batch normalization
GA: Genetic algorithm
GLCM: Gray-level co-occurrence matrix
LBP: Local binary pattern
NIR: Near-infrared
PC: Principal component
PCA: Principal component analysis
PLS_DA: Partial least squares discriminant analysis
ROI: Region of interest
SG: Savitzky-Golay
SPA: Successive projection algorithm
SNV: Standard normal variate
