# Supplementary material for: A CNN model for early detection of pepper Phytophthora blight using multispectral imaging, integrating spectral and textural information
Source: Plant Methods. 2024 Jul 29;20:115. doi: 10.1186/s13007-024-01239-7 (PMC11288097; doi:10.1186/s13007-024-01239-7)
Supplement: Supplementary file 1 — Additional file 1: Figure results after SPA feature extraction;wavelength extracted at 48 h;wavelength extracted at 60 h;wavelength extracted at 72 h;wavelength extracted at 84 h. Figure results after GA feature extraction;wavelength extracted at 48 h;wavelength extracted at 60 h;wavelength extracted at 72 h;wavelength extracted at 84 h. Table Results of feature extraction using different methods. [file 13007_2024_1239_MOESM1_ESM.docx]

**Appendix A**


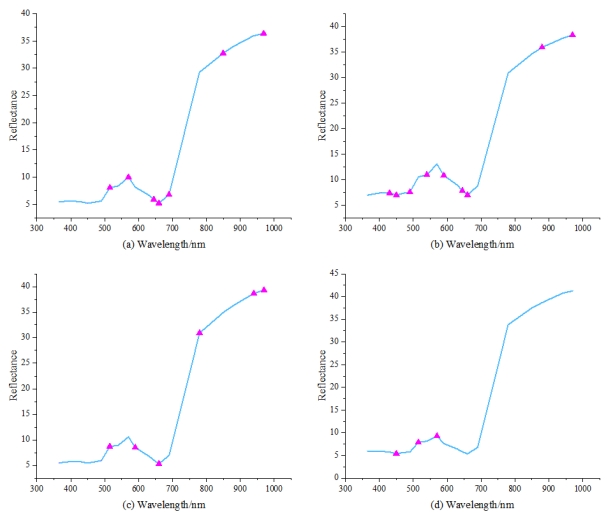


**Figure.A1** results after SPA feature extraction; (a) wavelength extracted at 48 h; (b) wavelength extracted at 60 h; (c) wavelength extracted at 72 h; (d) wavelength extracted at 84 h.


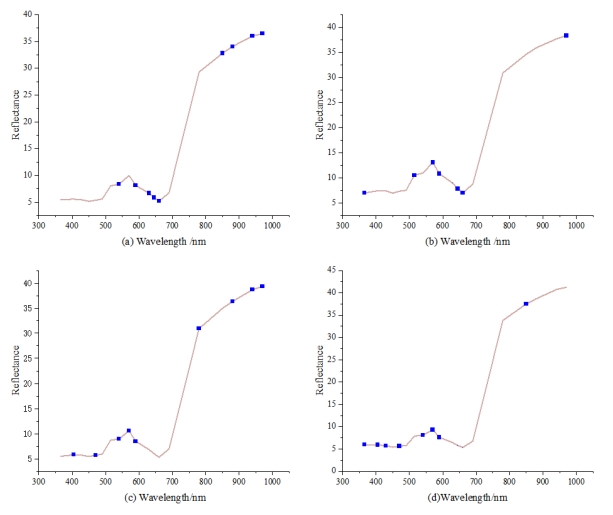


**Figure.A2** results after GA feature extraction; (a) wavelength extracted at 48 h; (b) wavelength extracted at 60 h; (c) wavelength extracted at 72 h; (d) wavelength extracted at 84 h.

**Table.A1** Results of feature extraction using different methods.

| Time/h | Method | Effective features |
| --- | --- | --- |
| 48 | SPA | 365 nm, 570 nm, 940 nm, 970 nm, and LBP58 |
|  | GA | 365 nm, 430 nm, 470 nm, 515 nm, 570 nm, 645 nm, 690 nm, 940 nm, 970 nm, LBP54, and LBP58 |
| 60 | SPA | 450 nm, 470 nm, 515 nm, 630nm, and LBP58 |
|  | GA | 365 nm, 405 nm, 430 nm, 450 nm, 470 nm, 540 nm, 630 nm, 660 nm, 690 nm, 850 nm, 940 nm, homogeneity, LBP4, LBP8, LBP40, LBP52, LBP56, LBP58, and LBP59 |
| 72 | SPA | 430 nm, 570 nm, contrast, correlation, and energy |
|  | GA | 430 nm, 515 nm, 630 nm, 645 nm, 690 nm, LBP4, LBP38, LBP50, LBP54, and LBP56 |
| 84 | SPA | 570nm and homogeneity |
|  | GA | 450 nm, 470 nm, 490 nm, 515 nm, 570 nm, 645 nm, contrast, homogeneity, LBP8, LBP18, LBP22, LBP50, LBP52, LBP54, LBP56, and LBP59 |
